# Investigation of the relationships between sports anxiety, positive thinking skills, and life satisfaction in male athletes

**DOI:** 10.3389/fpsyg.2024.1460257

**Published:** 2024-11-07

**Authors:** Ersan Tolukan, Aydiner Birsin Yildiz, Ibrahim Fatih Yenel, Ilimdar Yalcin, Leonard Stoica, Daniel-Andrei Iordan, Onu Ilie

**Affiliations:** ^1^Department of Sports Management, Faculty of Sport Sciences, Ankara Yildirim Beyazit University, Ankara, Türkiye; ^2^Department of Sports Management, Faculty of Sport Sciences, Gazi University, Ankara, Türkiye; ^3^Department of Coaching Education, Faculty of Sport Sciences, Bingol University, Bingol, Türkiye; ^4^Faculty of Physical Education and Sport, Department of Individual Sports and Physical Therapy, “Dunarea de Jos” University of Galati, Galati, Romania; ^5^Research Centre for Physical Therapy and Rehabilitation, “Dunarea de Jos” University of Galati, Romania; ^6^Faculty of Medical BioEngineering, Department of Biomedical Sciences, Grigore T. Popa University of Medicine and Pharmacy, Iasi, Romania; ^7^Department of Physiotherapy, Micromedica Clinic, Piatra Neamt, Romania

**Keywords:** athlete, life satisfaction, sport anxiety, sports psychology, positive thinking skill

## Abstract

Sports anxiety is an important obstacle for athletes’ performance, negatively affecting their life satisfaction levels. Positive thinking skills can contribute to overcoming such negative conditions. This study explored the relationships between sport anxiety, positive thinking skills, and life satisfaction in male athletes. A total of 338 male athletes participated voluntarily, using convenience sampling. The study employed a relational survey model, and data were collected through the Sports Anxiety Scale-2, Positive Thinking Skills Scale, and Life Satisfaction Scale. Analyses, including Pearson’s correlation, were performed using the JAMOVI program, with mediation analysis verified through bootstrapping. Results indicated a negative correlation between sport anxiety and life satisfaction, and a positive correlation between positive thinking skills and life satisfaction. Moreover, positive thinking skills were found to moderate the relationship between sport anxiety and life satisfaction. These insights underscore the value of developing positive thinking skills to help athletes reduce anxiety and enhance their life satisfaction. Therefore, incorporating strategies to foster these skills in training programs could be crucial for improving athletes’ overall wellbeing.

## Introduction

Mental health is a complex concept that must be understood as more than just the absence of mental illness. The World Health Organization (WHO) defines mental health as “a state of wellbeing in which individuals realize their own abilities, can cope with the normal stresses of life, work productively, and contribute to their community” (World Health Organization, 2001). Poor mental health is a risk factor for various diseases (Lombardo et al., 2018), and there is a strong inverse relationship between mental health and life satisfaction (Rissanen et al., 2013; Strine et al., 2009; Fergusson et al., 2015; Rissanen et al., 2011; Bray and Gunnell, 2006; Touburg and Veenhoven, 2015). Thus, life satisfaction emerges as a critical concept in health research. It is especially relevant for athletes, who face challenging life conditions due to factors such as high training loads and pressure to succeed. Life satisfaction has been shown to protect athletes from stress (Chen et al., 2017), yet it is also acknowledged that performance pressure can diminish life satisfaction in athletes (Felton and Jowett, 2015).

Anxiety, which is defined as a negative emotional state triggered by physiological arousal, is positively correlated with numerous adverse conditions and detrimentally affects athletic performance (World Health Organization, 2001; Lombardo et al., 2018). These findings also suggest that anxiety is inversely related to positive psychological states. While previous research has shown an inverse relationship between anxiety and life satisfaction (Ayten and Bakır, 2021; Sanioğlu et al., 2018; Kermen et al., 2016; Mahmoud et al., 2012; Beutel et al., 2010), the ongoing interest in understanding this relationship underscores the importance of further investigation, particularly in athletic populations.

Positive thinking skills have gained prominence in recent years as a key factor in maintaining and enhancing positive psychological states. Positive thinking is a cognitive process that creates hopeful images, develops optimistic ideas, finds positive solutions to problems, makes positive decisions, and produces a bright outlook on life in general, without ignoring realism. The ability to think positively is the ability to move toward a positive focus and interpretation, recognizing both the negative and positive aspects of circumstances. As individual differences in thinking patterns exist, providing evidence on the benefits of positive thinking is essential. Positive thinking serves as a critical source of motivation, enabling individuals to approach life with clarity (McGrath, 2024). Those who effectively employ positive thinking skills tend to face challenges optimistically and maintain control in situations that might otherwise provoke stress and anxiety. They also employ functional coping strategies that enable them to manage problems more effectively. Individuals with high levels of positive thinking report that their lives are progressing well, their goals are being met, and they have sufficient resources to cope (Carver and Scheier, 1998; Cantor et al., 1991). These conditions are often associated with increased effort and, consequently, enhanced performance. Previous studies indicate a linear relationship between positive thinking skills and positive experiences, success, and energy in various activities, as well as an inverse relationship between positive thinking and anxiety and stress (Yang et al., 2020).

While much research has been conducted on anxiety and life satisfaction in general populations, fewer studies have explored the moderating role of positive thinking skills in the context of sports, particularly among male athletes. This study aims to fill this gap by investigating the interplay between these psychological factors. Positive thinking skills are viewed as a key psychological resource that enables individuals to reframe stress-inducing situations and employ more adaptive coping strategies. Drawing on cognitive-behavioral theories, positive thinking skills are expected to act as a moderator, mitigating the negative effects of anxiety on life satisfaction.

Based on these findings, it is hypothesized that the relationship between sports anxiety and life satisfaction is particularly relevant in athletic settings, where overcoming challenging conditions is essential. Sports anxiety is expected to negatively affect life satisfaction, while positive thinking skills may serve as a moderating factor, reducing the negative impact of anxiety. In this context, the study tested a theoretical model in which positive thinking skills were included as a moderating variable in the relationship between sport anxiety and life satisfaction among male athletes. This study concentrated on male athletes to address the specific pressures and psychological challenges they face, such as societal expectations and performance demands, which may differ from those of female athletes. Focusing on this population allowed for a more in-depth exploration of how sports anxiety and positive thinking skills impact life satisfaction in male athletes. While the study is limited by its exclusive focus on males, it provides a crucial foundation for understanding these dynamics in male athletes, laying the groundwork for future research that can include female athletes or examine gender differences more explicitly. The results of this study are expected to contribute to the psychological literature on male athletes. For this reason, the study tested the moderating role of positive thinking skills in the indirect effect of sport anxiety on life satisfaction in male athletes. The hypotheses and theoretical model established in this context are given method section.

## Methods

### The research design

**Figure 1:**
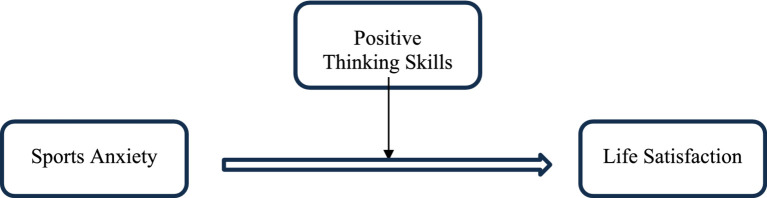
The theoretical model established within the scope of the research.

This research is a correlational study designed to explore a unique theoretical framework investigating the moderating role of positive thinking skills. The model includes three variables: sport anxiety, life satisfaction, and positive thinking skills. Positive thinking skills were treated as a moderating variable in the relationship between sport anxiety and life satisfaction. The hypotheses established in this context are outlined below, and the theoretical model developed and tested in the study is visualized in Figure 1.

*H1*: Sport anxiety has a negative relationship with life satisfaction.

*H2*: There is a positive relationship between positive thinking skills and life satisfaction.

*H3*: There is a negative relationship between positive thinking skills and sports anxiety.

*H4*: Positive thinking skills have a moderating role in the relationship between sport anxiety and life satisfaction.

### Participants

There is no consensus on the ideal number of participants required for statistical modeling. In general, a sample size of fewer than 100 is considered small, while a sample size of more than 200 is regarded as large (Bentler and Chou, 1987). Another approach considers the number of variables, where it is accepted that, for normally distributed data, a sample size at least 5 times the number of latent variables is sufficient (Kline, 2005). Based on this, 338 athletes were included in the study. The data used in the study were obtained from 338 male athletes with an average age of 22.5 ± 3.51, who constituted the study group voluntarily based on the convenience sampling method. It was determined that the sports age of male athletes in different branches (athletics, badminton, basketball, gymnastics, fitness, football, tennis, volleyball, swimming, and martial arts) was 7.47 ± 3.53.

### Research ethics

This research was conducted in accordance with the decision of Ankara Yildirim Beyazit University Ethics Committee was carried out in accordance with the authorization. In addition, the Helsinki Declaration was taken into consideration in the whole research process.

### Data collection tools

The data used in the study were collected through the Personal Information Form, Sports Anxiety Scale-2, Positive Thinking Skills Scale, and Life Satisfaction Scale. Sport Anxiety Scale-2 was developed by Smith et al. (2006) and adapted into Turkish by Karadağ and Aşçı (2020). The scale consists of 15 questions and has a 4-point Likert type. It has 4 subscales named as somatic anxiety, anxiety, and concentration. The Cronbach’s alpha coefficient of the Turkish version of the scale was determined as 0.89. Positive Thinking Skills Scale was developed by Bekhet and Zauszniewski (2013) and adapted into Turkish by Akın et al. (2015). The scale consists of 8 questions and has a 4-point Likert type. The Cronbach’s alpha coefficient of the Turkish version of the scale was determined as 0.90. Satisfaction with Life Scale was developed by Diener et al. (1985) and adapted into Turkish by Dağlı and Baysal (2016). The scale consists of 5 questions and is a 7-point Likert-type scale. The Cronbach’s alpha coefficient of the Turkish version of the scale was determined as 0.80. Some descriptive information of the male athletes constituting the study group of the research is given in Table 1.

**Table 1:** Construct validity and reliability results.

	Sport anxiety	Positive thinking skills	Life satisfaction
Minimum	15	7	7
Maximum	39	24	25
Mean	26.7	14.6	16.7
Standard deviation	5.71	4.59	5.43
Skewness	−0.132	0.130	−0.409
Kurtosis	−0.397	−0.393	−1.15
Cronbach’s α	0.868	0.905	0.946
McDonald’s ω	0.879	0.911	0.949

### Data collection

The data used in this study were voluntarily provided by the athletes after the research was introduced to them. Before participating, the athletes were thoroughly informed about the study’s purpose and scope. They were also told that they could withdraw from the study at any time without providing a reason. Additionally, they were assured that their responses would remain confidential and would not be shared with anyone outside the research team. The results would be reported solely within the context of this study, without disclosing any personal information. Athletes who consented to participate were then asked to complete the measurement tools.

### Statistical analysis

In this study, internal consistency was assessed using Cronbach’s Alpha coefficient, and reliability was evaluated according to DeVellis and Thorpe’s guidelines (DeVellis and Thorpe, 2021), ensuring that all items measured the same construct. Data distribution was examined through skewness, kurtosis values, and visual graphs. Based on George and Mallery’s criteria (George and Mallery, 2010), the data showed a normal distribution, justifying the use of parametric tests in the analysis (Table 1).

Pearson correlation coefficients were calculated to test Hypothesis 1, which examined the relationships between the main variables. According to Cohen’s guidelines, correlations were classified as small (*r* = 0.10–0.29), medium (*r* = 0.30–0.49), and large (*r* ≥ 0.50) (DeVellis and Thorpe, 2021), providing a clearer understanding of the strength of these relationships. To test Hypothesis 2, the JAMOVI medmod bootstrap estimation method (5.000 samples) was employed to assess the moderating role of positive thinking skills in the relationship between sports anxiety and life satisfaction. Moderation effects were considered significant when the bootstrap confidence intervals (CI) did not include zero, indicating a meaningful moderating influence. All analyses were conducted using JAMOVI (version 2.5.2.0), with a significance level set at *p* < 0.05.

## Results

### Descriptive statistics

Table 1 presents the descriptive statistics derived from the study’s data. The Cronbach’s Alpha values for all scales were above 0.86, indicating a high level of reliability in the responses (Diener et al., 1985). Additionally, skewness and kurtosis values fell within the acceptable range of ±1.5, suggesting that the data adhered to a normal distribution. When examining the participants’ mean scores across the scales, it can be inferred that they exhibit average levels in the relevant characteristics.

### Correlation analysis results

Table 2 shows the Pearson correlation coefficients calculated to determine the relationships between sports anxiety, positive thinking skills, and life satisfaction. The analysis revealed a weak negative relationship between sports anxiety and positive thinking skills (*r* = −0.184, *p* < 0.001), and a moderate negative relationship between sports anxiety and life satisfaction (*r* = −0.439, *p* < 0.001). Additionally, a moderate positive relationship was found between positive thinking skills and life satisfaction (*r* = 0.462, *p* < 0.001). These results indicate three key relationships. First, there is a negative relationship between sport anxiety and life satisfaction, meaning that as sport anxiety increases, life satisfaction decreases. Second, there is an inverse relationship between positive thinking skills and sport anxiety, suggesting that athletes with higher positive thinking skills tend to experience lower levels of sport anxiety. Third, a positive relationship was found between positive thinking skills and life satisfaction, indicating that as positive thinking skills improve, so does life satisfaction.

**Table 2:** Correlation analysis results.

		SA	PTS	LS
Sport Anxiety ^SA^	p			
	r			
Positive Thinking Skills ^PTS^	p	−0.184**		
	r	<0.001		
Life Satisfaction ^LS^	p	−0.439**	0.462**	
	r	<0.001	<0.001	

### Moderation analysis results

Table 3 presents the results of the moderation analysis. When the results were examined, it was determined that sports anxiety, identified as the independent variable, negatively affected life satisfaction, identified as the dependent variable in the model (Est = −0.284, SE = 0.047, *Z* = −6.05, *p* < 0.001). Positive thinking skills, identified as the moderator variable, had a positive effect (Est = 0.514, SE = 0.0551, *Z* = 9.44, *p* < 0.001). Furthermore, the moderating effect of positive thinking skills on the interaction between sports anxiety and life satisfaction was found to be significant (Est = 0.026, SE = 0.0086, *Z* = 3.03, *p* = 0.002).

**Table 3:** Results of moderation analysis.

	Estimate	SE	95% C.I.		*Z*	*p*
			LLCI	ULCI		
Sport Anxiety	−0.284	0.0470	−0.372	−0.186	−6.05	<0.001
Positive Thinking Skills	0.514	0.0551	−0.411	0.628	9.44	<0.001
Life Satisfaction ✻ Positive Thinking Skills	0.026	0.0086	0.0093	0.043	3.03	0.002

SE, standardized estimate; C.I, confidence interval; LLCI, lower confidence interval; ULCI, upper confidence interval.

Table 4 shows the detailed analysis results of the moderating effect. When the results are analyzed, it is seen that positive thinking skills were found to be moderate in the details of the moderating effect (Est = −0.284, *p* = <0.001), low (Est = −0.404, *p* < 0.001) and high (Est = −0.164, *p* = 0.011).

**Table 4:** Simple slope analysis results showing moderation effects.

	Estimate	SE	95% C.I.		*Z*	*p*
			LLCI	ULCI		
Average	−0.284	0.0476	−0.374	−0.185	−5.96	<0.001
Low (-1SD)	−0.404	0.0590	−0.514	−0.280	−6.84	<0.001
High (+1SD)	−0.164	0.0645	−0.288	−0.034	−2.54	0.011

SE, standardized estimate; C.I, confidence interval; LLCI, lower confidence interval; ULCI, upper confidence interval.

**Figure 2:**
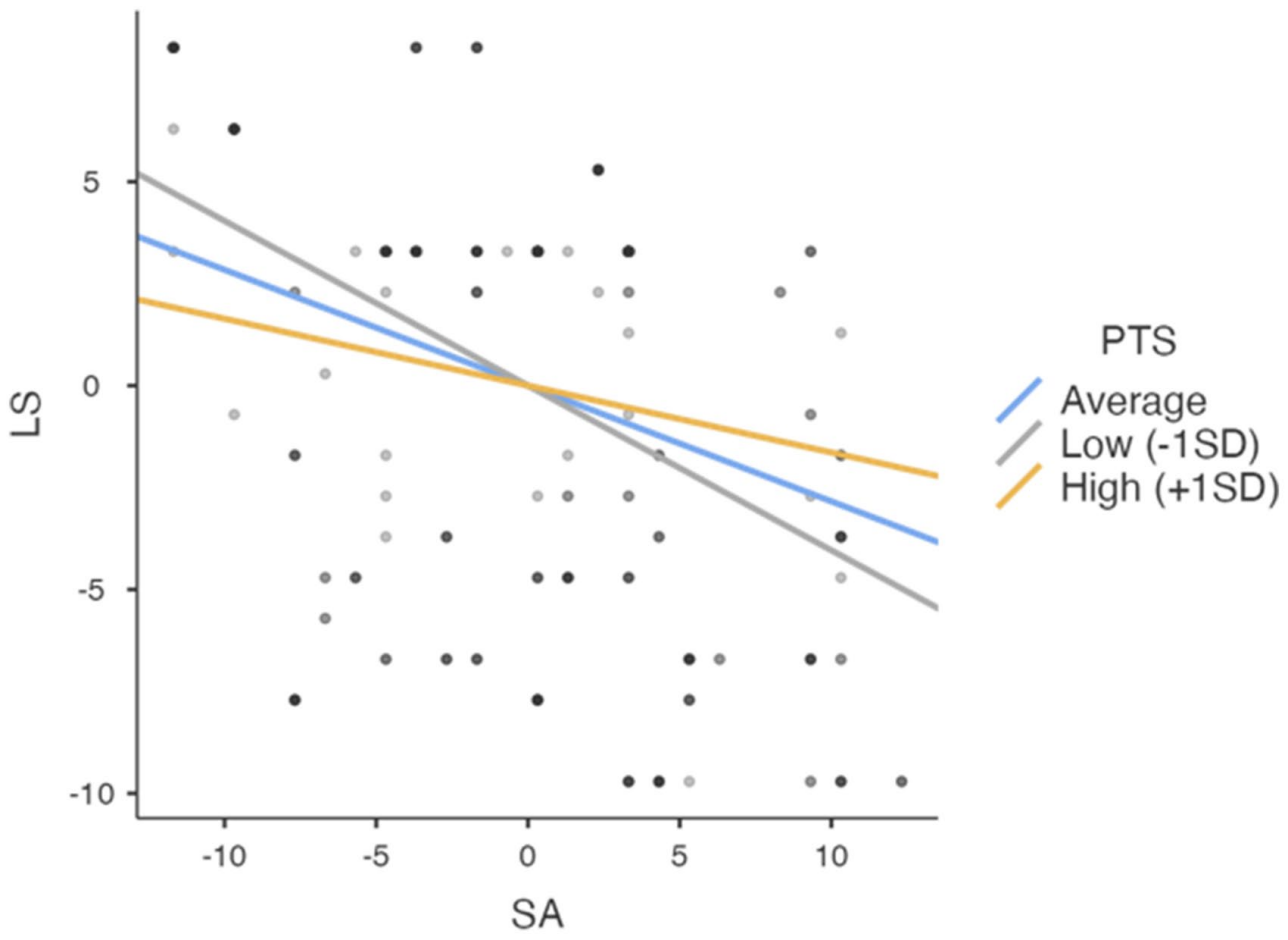
Simple slope plot.

According to the results of simple slope analysis, the effects of the regulator variable were shown in Figure 2.

When Tables 3, 4 and Figure 2 are examined together, the moderation analysis indicates that positive thinking skills significantly moderate the relationship between sport anxiety and life satisfaction. Specifically, athletes with higher positive thinking skills experience a weaker negative impact of sport anxiety on life satisfaction. This suggests that developing positive thinking skills may help mitigate the adverse effects of sport anxiety on life satisfaction.

## Discussion

Although numerous studies have explored anxiety and life satisfaction in general populations, there is a lack of research examining the moderating role of positive thinking skills specifically in male athletes. This study was conducted to address this gap in order to alleviate this issue, with the hypotheses that sport anxiety is negatively related to life satisfaction, positive thinking skills are positively related to life satisfaction, and positive thinking skills are negatively related to sport anxiety. Additionally, the study tested a theoretical model in which positive thinking skills serve as a moderator in the relationship between sport anxiety and life satisfaction. The findings of this study provide valuable insights into the relationships between sports anxiety, positive thinking skills, and life satisfaction specifically among male athletes. The results emphasize how sports anxiety negatively impacts life satisfaction, while positive thinking skills act as a protective factor. These findings are consistent with recent literature focusing on male athletes and their psychological wellbeing.

The results first revealed a negative relationship between sports anxiety and life satisfaction (Table 2). With this result, the first hypothesis was confirmed. This finding means that there will be a decrease in life satisfaction with an increase in sport anxiety. Athletes often find it difficult to achieve life satisfaction due to the high physical and mental demands of training and the pressure to succeed. The findings of this study align with previous research indicating a negative correlation between anxiety and life satisfaction (Beutel et al., 2010; Yıldırım and Özgökçe, 2023; Perveen et al., 2023; Surujlal et al., 2013). This suggests that anxiety is not only a performance issue but also a factor that significantly affects broader aspects of wellbeing.

Secondly, the study found a positive relationship between positive thinking skills and life satisfaction (Table 2). With this result, the second hypothesis was confirmed. This finding means that an increase in positive thinking skills will lead to an increase in life satisfaction. Positive thinking skills can help athletes cope with adversities, thus contributing to higher life satisfaction. This finding is supported by previous research, which similarly identifies a positive association between positive thinking and life satisfaction (Khan and Siddiqui, 2021; Taherkhani et al., 2023; Cohn et al., 2009). The negative relationship between sports anxiety and positive thinking skills further supports the idea that male athletes who engage in positive cognitive strategies experience less anxiety. These results support the cognitive-behavioral approach, which posits that positive thinking can help athletes reframe negative experiences and reduce anxiety.

The study also identified a negative correlation between positive thinking skills and sports anxiety (Table 2). With this result, the third hypothesis was confirmed. This finding means that there will be a decrease in sport anxiety with an increase in positive thinking skills. It is well-established that individuals with greater emotional variability, often associated with negative thinking, tend to experience poorer psychological health (Gruber et al., 2013). In contrast, individuals who possess strong positive thinking skills are more capable of overcoming life challenges by replacing negative thoughts with constructive ones, as supported by prior studies (Yue et al., 2022; Andrade, 2019). The positive correlation between positive thinking skills and life satisfaction underscores the importance of these skills in fostering wellbeing among male athletes. This highlights that positive thinking not only helps mitigate anxiety but also contributes to a more fulfilling life, aligning with the broaden-and-build theory of positive emotions.

Lastly, the findings showed that positive thinking skills have a moderating effect on the relationship between sports anxiety and life satisfaction (Tables 3, 4). With this result, the fourth hypothesis was confirmed. This finding means that the relationship between sport anxiety and life satisfaction can be differentiated by positive thinking skills. In addition to the direct influence of positive thinking on life satisfaction, previous studies suggest that it can also have an indirect effect. For instance, Cohn et al. (2009) provide evidence that positive thinking enhances life satisfaction by fostering resilience. Sanchez and Vazquez (2014) demonstrated that positive emotions can mediate the effect of life satisfaction on attention to positive stimuli, such as happy faces. Similarly, Lightsey and Boyraz (2011) found that positive affect mediates the relationship between positive cognitions and both meaning in life and life satisfaction. The moderation analysis in this study suggests that positive thinking skills significantly weaken the negative impact of sports anxiety on life satisfaction. This is particularly relevant for male athletes who often face high performance pressure. These findings are in line with previous studies, which suggest that individuals with a positive mental framework are more likely to interpret negative events in a favorable light (Zaidel et al., 2021; Xu et al., 2020). This leads to increased life satisfaction, which serves as a vital mechanism for athletes to prevent stress (Chen et al., 2017). This reinforces the notion that psychological resilience, fostered through positive thinking, can be a crucial tool in maintaining life satisfaction despite the challenges of competitive sports.

In conclusion, this study explored the relationships between sport anxiety, positive thinking skills, and life satisfaction in male athletes, addressing a significant gap in the literature. The findings identified three key relationships: a negative correlation between sport anxiety and life satisfaction, an inverse relationship between positive thinking skills and sport anxiety, and a positive correlation between positive thinking skills and life satisfaction. Additionally, positive thinking skills were found to moderate the negative effects of sport anxiety on life satisfaction, emphasizing their protective role.

These results contribute to the growing body of evidence in sports psychology, underscoring the critical role of cognitive strategies such as positive thinking in managing competitive stress. Athletes with higher levels of positive thinking skills experience reduced anxiety and enhanced psychological wellbeing, which in turn improves their life satisfaction. The theoretical model developed in this study suggests that interventions aimed at enhancing these cognitive skills could have practical benefits for reducing sport-related anxiety and promoting overall life satisfaction in athletes. These findings indicate that coaches, sports psychologists, and trainers should implement these strategies to improve athletes’ mental resilience and wellbeing.

## Limitations

This study has several limitations. The fact that the data were collected exclusively from male athletes limits the generalizability of the findings to the broader athletic population or to female athletes. As the study employed a cross-sectional design, it was not possible to determine causal relationships between the variables. The reliance on self-report measures introduces the potential for response bias in participants’ answers. Additionally, the limited sample size and diversity restrict the generalizability of the results to athletes from different sports and age groups. The use of convenience sampling further limits the representativeness of the sample, making it difficult to generalize the findings to a wider population. The study’s specific cultural context also suggests that the findings may not be applicable to athletes in other cultural settings. Furthermore, the absence of any control variables in the analyses is another limitation that should be considered when interpreting the results. Finally, the descriptive nature of the research indicates that the findings should be interpreted with caution, as further in-depth analyses are required.

## Conclusion

### Theoretical implications

This study enriches our understanding of the effectiveness of positive thinking skills in overcoming sport anxiety. By establishing a clear link between sport anxiety and life satisfaction, it highlights the critical role of positive emotions in promoting life satisfaction among athletes. These findings suggest that overcoming sport anxiety is essential for athletes to achieve life satisfaction. Furthermore, the evidence that positive thinking acts as a moderator in this relationship offers a fresh perspective on how psychological skills can influence wellbeing. The study provides valuable insights that may guide future research in exploring the mechanisms by which positive thinking skills help prevent sport anxiety and enhance life satisfaction. These contributions expand the theoretical framework surrounding sport psychology, emphasizing the importance of psychological resilience and cognitive strategies in maintaining emotional and psychological wellbeing in demanding athletic environments.

### Practical implications

This highlights the importance of integrating positive thinking skills into the training and development programs of athletes. This study underlines the benefits of having positive thinking skills to increase life satisfaction levels for athletes and suggests applicable strategies for both athletes and coaches. Athletes should strive to overcome their sport anxiety in order to increase their life satisfaction levels, with the awareness that their life satisfaction levels can affect their performance. Based on the results of the research, it can be said that focusing on positive thinking skills may be useful in this regard. With the same perspective, coaches should create mechanisms to support their athletes in developing positive thinking skills to overcome their sport anxiety.

Coaches and sports psychologists should prioritize training athletes in mental resilience and cognitive strategies that foster positive thinking. In particular, techniques that enhance focus, stress management, and emotional regulation could be essential in reducing sport anxiety and enhancing life satisfaction. Additionally, developing effective anxiety management strategies is critical. Coaches can implement relaxation techniques, mindfulness practices, and cognitive restructuring to help athletes cope with competitive pressure. Regular psychological assessments and monitoring of athletes’ wellbeing are also recommended, as this will allow early detection of potential psychological issues. The findings further emphasize the role of positive thinking in not only improving individual performance but also creating a supportive team environment. Lastly, enhancing athletes’ overall life satisfaction through programs focused on career development, social support, and personal growth will likely contribute to their long-term psychological health and athletic success.

### Future research

Future research should investigate the effects of various factors, such as athletic mental energy, emotional intelligence, and the coach-athlete relationship, on overcoming sport anxiety and improving life satisfaction. Exploring the interplay between these factors and how they influence athletes’ mental health and performance can provide valuable insights. Additionally, it would be beneficial to examine the role of social support systems, including family, peers, and sports communities, in mitigating sport anxiety and enhancing life satisfaction among athletes. Longitudinal research is crucial for a more nuanced understanding of these relationships. Such studies would allow researchers to observe changes in sport anxiety and life satisfaction over time, providing a clearer picture of causality. Furthermore, examining diverse populations, including female athletes and those from various sports backgrounds, will contribute to a more comprehensive understanding of the dynamics at play. This can lead to the development of tailored interventions that address specific needs and challenges faced by different athlete groups.

Finally, future studies could also focus on the implementation and effectiveness of training programs designed to enhance positive thinking skills and other psychological resilience factors in athletes. Understanding how these interventions can be integrated into regular training routines may provide practical solutions to enhance athletes’ overall wellbeing and performance.

## Data Availability

The raw data supporting the conclusions of this article will be made available by the authors, without undue reservation.
